# [*N*′-(5-Bromo-2-oxidobenzyl­idene-κ*O*)-3-hydr­oxy-2-naphthohydrazidato-κ^2^ *N*′,*O*]dimethyl­tin(IV)

**DOI:** 10.1107/S1600536810001133

**Published:** 2010-01-16

**Authors:** See Mun Lee, Hapipah Mohd Ali, Kong Mun Lo

**Affiliations:** a Department of Chemistry, University of Malaya, 50603 Kuala Lumpur, Malaysia

## Abstract

The Sn^IV^ atom in the title compound, [Sn(CH_3_)_2_(C_18_H_11_BrN_2_O_3_)], shows a distorted *cis*-C_2_NO_2_Sn trigonal-bipyramidal coordination geometry, with an axial O—Sn—O angle of 155.27 (9)°. The presence of an intra­molecular O—H⋯N hydrogen bond between the amido N atom and hydr­oxy H atom in the Schiff base ligand helps to stabilize the overall mol­ecular structure.

## Related literature

For related structures, see Lee *et al.* (2009*a* ▶,*b* ▶). For similar hydrazone dianions acting as *O*,*N*,*O*′-chelate ligands to tin in organotin compounds, see: Labib *et al.* (1996 ▶); Samanta *et al.* (2007 ▶).
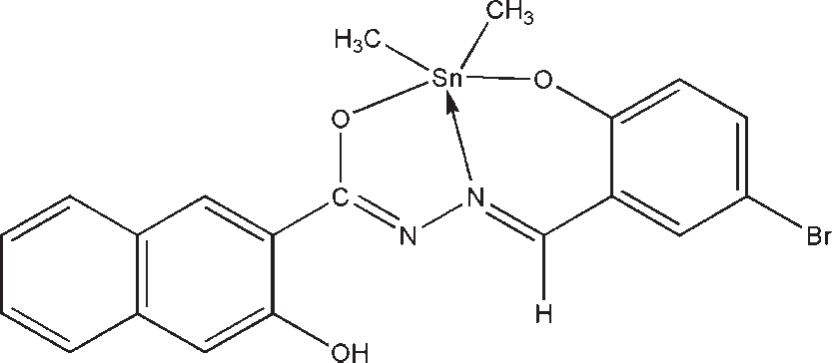


## Experimental

### 

#### Crystal data

[Sn(CH_3_)_2_(C_18_H_11_BrN_2_O_3_)]
*M* *_r_* = 531.96
Triclinic, 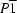

*a* = 6.8662 (5) Å
*b* = 11.7998 (9) Å
*c* = 11.9365 (9) Å
α = 87.464 (1)°
β = 76.128 (1)°
γ = 81.213 (1)°
*V* = 927.84 (12) Å^3^
*Z* = 2
Mo *K*α radiation
μ = 3.55 mm^−1^
*T* = 145 K
0.39 × 0.37 × 0.09 mm

#### Data collection

Bruker APEXII CCD area-detector diffractometer
Absorption correction: multi-scan (*SADABS*; Sheldrick, 1996 ▶) *T*_min_ = 0.338, *T*_max_ = 0.740
5350 measured reflections
4028 independent reflections
3703 reflections with *I* > 2σ(*I*)
*R*_int_ = 0.016

#### Refinement

*R*[*F* ^2^ > 2σ(*F* ^2^)] = 0.024
*wR*(*F* ^2^) = 0.079
*S* = 1.14
4028 reflections
245 parameters
1 restraint
H-atom parameters constrained
Δρ_max_ = 0.63 e Å^−3^
Δρ_min_ = −0.76 e Å^−3^

### 

Data collection: *APEX2* (Bruker, 2008 ▶); cell refinement: *SAINT* (Bruker, 2008 ▶0); data reduction: *SAINT*; program(s) used to solve structure: *SHELXS97* (Sheldrick, 2008 ▶); program(s) used to refine structure: *SHELXL97* (Sheldrick, 2008 ▶); molecular graphics: *X-SEED* (Barbour, 2001 ▶); software used to prepare material for publication: *publCIF* (Westrip, 2010 ▶).

## Supplementary Material

Crystal structure: contains datablocks I, global. DOI: 10.1107/S1600536810001133/sj2691sup1.cif

Structure factors: contains datablocks I. DOI: 10.1107/S1600536810001133/sj2691Isup2.hkl

Additional supplementary materials:  crystallographic information; 3D view; checkCIF report

## Figures and Tables

**Table 1:** Hydrogen-bond geometry (Å, °)

*D*—H⋯*A*	*D*—H	H⋯*A*	*D*⋯*A*	*D*—H⋯*A*
O3—H3⋯N2	0.84	1.88	2.611 (4)	144

**Fig. 1.**
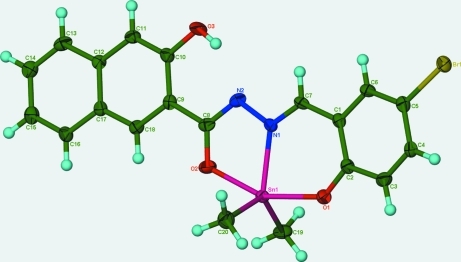
The molecular structure of [N'-(5-bromo-2-oxidobenzylidene-κO)-3-hydroxy-2- naphthohydrazidato-κ2N',O]dimethyltin(IV) showing 70% probability displacement ellipsoids and the atom numbering. Hydrogen atoms are drawn as spheres of arbitrary radius.

## supplementary crystallographic information

### Experimental

The Schiff base ligand was prepared by the condensation reaction of 3-hydroxy-2-naphthoyl hydrazide with 5-bromosalicylaldehyde. The title compound was prepared by refluxing the Schiff base (0.74 g, 2.0 mmol) with dimethyltin oxide (0.32 g, 2.0 mmol) in toluene for 6 h. The solution was filtered and left for recrystallization for a week during which yellow crystals were obtained.

### Refinement

All H-atoms were positioned geometrically and refined using a riding model with d(C-H) = 0.95Å,  U_iso_=1.2U_eq_ (C) for aromatic 0.98Å,  U_iso_ = 1.5U_eq_ (C) for CH_3_ atoms and 0.84Å, U_iso_ = 1.5U_eq_ (O) for the OH group.

### Crystal data

**Table:**

[Sn(CH_3_)_2_(C_18_H_11_BrN_2_O_3_)]	*Z* = 2
*M**_r_* = 531.96	*F*(000) = 520
Triclinic, *P*1	*D*_x_ = 1.904 Mg m^−^^3^
Hall symbol: -P 1	Mo *K*α radiation, λ = 0.71073 Å
*a* = 6.8662 (5) Å	Cell parameters from 3900 reflections
*b* = 11.7998 (9) Å	θ = 2.5–30.6°
*c* = 11.9365 (9) Å	µ = 3.55 mm^−^^1^
α = 87.464 (1)°	*T* = 145 K
β = 76.128 (1)°	Plate, yellow
γ = 81.213 (1)°	0.39 × 0.37 × 0.09 mm
*V* = 927.84 (12) Å^3^	

### Data collection

**Table:**

Bruker APEXII CCD area-detector diffractometer	4028 independent reflections
Radiation source: fine-focus sealed tube	3703 reflections with *I* > 2σ(*I*)
graphite	*R*_int_ = 0.016
ω scans	θ_max_ = 27.5°, θ_min_ = 1.8°
Absorption correction: multi-scan (*SADABS*; Sheldrick, 1996)	*h* = −8→8
*T*_min_ = 0.338, *T*_max_ = 0.740	*k* = −10→15
5350 measured reflections	*l* = −15→15

### Refinement

**Table:**

Refinement on *F*^2^	Primary atom site location: structure-invariant direct methods
Least-squares matrix: full	Secondary atom site location: difference Fourier map
*R*[*F*^2^ > 2σ(*F*^2^)] = 0.024	Hydrogen site location: inferred from neighbouring sites
*wR*(*F*^2^) = 0.079	H-atom parameters constrained
*S* = 1.14	*w* = 1/[σ^2^(*F*_o_^2^) + (0.0316*P*)^2^ + 1.8608*P*] where *P* = (*F*_o_^2^ + 2*F*_c_^2^)/3
4028 reflections	(Δ/σ)_max_ = 0.001
245 parameters	Δρ_max_ = 0.63 e Å^−^^3^
1 restraint	Δρ_min_ = −0.75 e Å^−^^3^

### Special details

**Table:**

Geometry. All e.s.d.'s (except the e.s.d. in the dihedral angle between two l.s. planes) are estimated using the full covariance matrix. The cell e.s.d.'s are taken into account individually in the estimation of e.s.d.'s in distances, angles and torsion angles; correlations between e.s.d.'s in cell parameters are only used when they are defined by crystal symmetry. An approximate (isotropic) treatment of cell e.s.d.'s is used for estimating e.s.d.'s involving l.s. planes.
Refinement. Refinement of *F*^2^ against ALL reflections. The weighted *R*-factor *wR* and goodness of fit *S* are based on *F*^2^, conventional *R*-factors *R* are based on *F*, with *F* set to zero for negative *F*^2^. The threshold expression of *F*^2^ > σ(*F*^2^) is used only for calculating *R*-factors(gt) *etc*. and is not relevant to the choice of reflections for refinement. *R*-factors based on *F*^2^ are statistically about twice as large as those based on *F*, and *R*- factors based on ALL data will be even larger.

### Fractional atomic coordinates and isotropic or equivalent isotropic displacement parameters (Å^2^)

**Table:**

	*x*	*y*	*z*	*U*_iso_*/*U*_eq_	
Sn1	0.75049 (3)	0.561594 (19)	0.670242 (18)	0.01412 (8)	
Br1	0.57215 (5)	−0.06224 (3)	0.64595 (3)	0.02011 (9)	
N1	0.5307 (4)	0.4692 (2)	0.7889 (2)	0.0141 (5)	
N2	0.3995 (4)	0.5332 (3)	0.8788 (2)	0.0172 (6)	
O1	0.8546 (4)	0.4001 (2)	0.5966 (2)	0.0202 (5)	
O2	0.5723 (4)	0.6809 (2)	0.7987 (2)	0.0228 (5)	
O3	0.1095 (4)	0.5698 (2)	1.0656 (2)	0.0219 (5)	
H3	0.1750	0.5344	1.0054	0.033*	
C1	0.6138 (5)	0.2791 (3)	0.6996 (3)	0.0141 (6)	
C2	0.7792 (5)	0.3027 (3)	0.6092 (3)	0.0152 (6)	
C3	0.8681 (5)	0.2152 (3)	0.5277 (3)	0.0188 (7)	
H3A	0.9776	0.2288	0.4655	0.023*	
C4	0.7999 (5)	0.1105 (3)	0.5364 (3)	0.0158 (6)	
H4	0.8593	0.0541	0.4790	0.019*	
C5	0.6435 (5)	0.0871 (3)	0.6295 (3)	0.0164 (6)	
C6	0.5524 (5)	0.1702 (3)	0.7093 (3)	0.0154 (6)	
H6	0.4459	0.1540	0.7723	0.019*	
C7	0.5032 (5)	0.3630 (3)	0.7853 (3)	0.0150 (6)	
H7	0.3999	0.3379	0.8450	0.018*	
C8	0.4332 (5)	0.6410 (3)	0.8761 (3)	0.0164 (6)	
C9	0.3027 (5)	0.7184 (3)	0.9668 (3)	0.0151 (6)	
C10	0.1483 (5)	0.6797 (3)	1.0581 (3)	0.0147 (6)	
C11	0.0383 (5)	0.7555 (3)	1.1419 (3)	0.0154 (6)	
H11	−0.0602	0.7291	1.2037	0.018*	
C12	0.0673 (5)	0.8711 (3)	1.1392 (3)	0.0139 (6)	
C13	−0.0460 (5)	0.9515 (3)	1.2244 (3)	0.0189 (7)	
H13	−0.1429	0.9267	1.2880	0.023*	
C14	−0.0175 (5)	1.0647 (3)	1.2160 (3)	0.0190 (7)	
H14	−0.0962	1.1174	1.2732	0.023*	
C15	0.1279 (5)	1.1033 (3)	1.1232 (3)	0.0192 (7)	
H15	0.1469	1.1816	1.1186	0.023*	
C16	0.2407 (5)	1.0289 (3)	1.0405 (3)	0.0164 (6)	
H16	0.3376	1.0558	0.9782	0.020*	
C17	0.2153 (5)	0.9112 (3)	1.0461 (3)	0.0138 (6)	
C18	0.3312 (5)	0.8320 (3)	0.9627 (3)	0.0152 (6)	
H18	0.4322	0.8573	0.9017	0.018*	
C19	1.0295 (5)	0.5826 (3)	0.7067 (3)	0.0201 (7)	
H19A	1.0478	0.5367	0.7748	0.030*	
H19B	1.0291	0.6637	0.7216	0.030*	
H19C	1.1409	0.5572	0.6405	0.030*	
C20	0.6241 (5)	0.6376 (3)	0.5344 (3)	0.0227 (7)	
H20A	0.4933	0.6117	0.5391	0.034*	
H20B	0.7165	0.6150	0.4601	0.034*	
H20C	0.6040	0.7213	0.5412	0.034*	

### Atomic displacement parameters (Å^2^)

**Table:**

	*U*^11^	*U*^22^	*U*^33^	*U*^12^	*U*^13^	*U*^23^
Sn1	0.01425 (12)	0.01281 (13)	0.01497 (12)	−0.00358 (8)	−0.00180 (8)	−0.00023 (8)
Br1	0.02169 (17)	0.01283 (18)	0.02579 (18)	−0.00506 (13)	−0.00342 (14)	−0.00316 (13)
N1	0.0149 (12)	0.0107 (13)	0.0174 (13)	−0.0042 (10)	−0.0034 (10)	0.0001 (10)
N2	0.0154 (13)	0.0220 (15)	0.0131 (12)	−0.0028 (11)	−0.0010 (10)	−0.0018 (11)
O1	0.0197 (12)	0.0140 (12)	0.0244 (12)	−0.0038 (9)	0.0011 (10)	−0.0017 (10)
O2	0.0216 (12)	0.0197 (13)	0.0232 (12)	−0.0073 (10)	0.0054 (10)	−0.0029 (10)
O3	0.0292 (13)	0.0127 (12)	0.0214 (12)	−0.0114 (10)	0.0050 (10)	−0.0056 (9)
C1	0.0160 (14)	0.0122 (15)	0.0151 (14)	−0.0024 (12)	−0.0058 (12)	−0.0001 (12)
C2	0.0162 (15)	0.0134 (16)	0.0172 (15)	−0.0026 (12)	−0.0061 (12)	0.0000 (12)
C3	0.0164 (15)	0.0223 (18)	0.0160 (15)	−0.0008 (13)	−0.0022 (12)	0.0031 (13)
C4	0.0175 (15)	0.0133 (16)	0.0168 (15)	−0.0006 (12)	−0.0051 (12)	−0.0014 (12)
C5	0.0173 (15)	0.0141 (16)	0.0194 (16)	−0.0024 (12)	−0.0075 (12)	−0.0002 (13)
C6	0.0146 (14)	0.0159 (16)	0.0171 (15)	−0.0062 (12)	−0.0042 (12)	0.0015 (12)
C7	0.0157 (14)	0.0140 (16)	0.0168 (15)	−0.0063 (12)	−0.0049 (12)	0.0033 (12)
C8	0.0131 (14)	0.0223 (18)	0.0143 (15)	−0.0037 (12)	−0.0040 (12)	0.0021 (13)
C9	0.0151 (14)	0.0150 (16)	0.0157 (15)	−0.0012 (12)	−0.0051 (12)	−0.0013 (12)
C10	0.0181 (15)	0.0119 (15)	0.0155 (15)	−0.0045 (12)	−0.0047 (12)	−0.0025 (12)
C11	0.0154 (14)	0.0174 (17)	0.0139 (14)	−0.0057 (12)	−0.0024 (12)	−0.0015 (12)
C12	0.0139 (14)	0.0144 (16)	0.0149 (14)	−0.0036 (12)	−0.0050 (11)	−0.0022 (12)
C13	0.0145 (15)	0.0284 (19)	0.0137 (15)	−0.0037 (13)	−0.0029 (12)	0.0001 (13)
C14	0.0202 (16)	0.0201 (18)	0.0178 (16)	−0.0024 (13)	−0.0059 (13)	−0.0044 (13)
C15	0.0207 (16)	0.0173 (17)	0.0206 (16)	−0.0011 (13)	−0.0073 (13)	−0.0035 (13)
C16	0.0182 (15)	0.0120 (16)	0.0202 (16)	−0.0047 (12)	−0.0051 (12)	−0.0025 (12)
C17	0.0162 (14)	0.0134 (15)	0.0125 (14)	−0.0016 (12)	−0.0049 (11)	−0.0024 (12)
C18	0.0147 (14)	0.0176 (17)	0.0132 (14)	−0.0020 (12)	−0.0035 (11)	−0.0007 (12)
C19	0.0173 (15)	0.0249 (19)	0.0193 (16)	−0.0090 (14)	−0.0031 (13)	−0.0007 (14)
C20	0.0225 (17)	0.0223 (19)	0.0238 (17)	−0.0015 (14)	−0.0079 (14)	0.0017 (14)

### Geometric parameters (Å, °)

**Table:**

Sn1—O1	2.084 (2)	C8—C9	1.477 (5)
Sn1—C19	2.116 (3)	C9—C18	1.381 (5)
Sn1—C20	2.120 (3)	C9—C10	1.438 (4)
Sn1—O2	2.143 (2)	C10—C11	1.372 (5)
Sn1—N1	2.194 (3)	C11—C12	1.406 (5)
Br1—C5	1.890 (3)	C11—H11	0.9500
N1—C7	1.300 (4)	C12—C13	1.421 (5)
N1—N2	1.390 (4)	C12—C17	1.432 (4)
N2—C8	1.324 (5)	C13—C14	1.375 (5)
O1—C2	1.320 (4)	C13—H13	0.9500
O2—C8	1.290 (4)	C14—C15	1.412 (5)
O3—C10	1.358 (4)	C14—H14	0.9500
O3—H3	0.8400	C15—C16	1.360 (5)
C1—C6	1.404 (4)	C15—H15	0.9500
C1—C2	1.419 (4)	C16—C17	1.422 (5)
C1—C7	1.446 (5)	C16—H16	0.9500
C2—C3	1.413 (5)	C17—C18	1.405 (4)
C3—C4	1.377 (5)	C18—H18	0.9500
C3—H3A	0.9500	C19—H19A	0.9800
C4—C5	1.399 (5)	C19—H19B	0.9800
C4—H4	0.9500	C19—H19C	0.9800
C5—C6	1.367 (5)	C20—H20A	0.9800
C6—H6	0.9500	C20—H20B	0.9800
C7—H7	0.9500	C20—H20C	0.9800
			
O1—Sn1—C19	95.10 (12)	C18—C9—C8	118.2 (3)
O1—Sn1—C20	97.09 (13)	C10—C9—C8	122.5 (3)
C19—Sn1—C20	127.84 (14)	O3—C10—C11	118.6 (3)
O1—Sn1—O2	155.27 (9)	O3—C10—C9	122.3 (3)
C19—Sn1—O2	94.33 (12)	C11—C10—C9	119.2 (3)
C20—Sn1—O2	95.04 (13)	C10—C11—C12	121.9 (3)
O1—Sn1—N1	83.01 (10)	C10—C11—H11	119.0
C19—Sn1—N1	121.63 (12)	C12—C11—H11	119.0
C20—Sn1—N1	110.12 (12)	C11—C12—C13	123.0 (3)
O2—Sn1—N1	72.59 (10)	C11—C12—C17	119.2 (3)
C7—N1—N2	115.3 (3)	C13—C12—C17	117.8 (3)
C7—N1—Sn1	128.8 (2)	C14—C13—C12	121.0 (3)
N2—N1—Sn1	115.9 (2)	C14—C13—H13	119.5
C8—N2—N1	112.2 (3)	C12—C13—H13	119.5
C2—O1—Sn1	133.1 (2)	C13—C14—C15	120.6 (3)
C8—O2—Sn1	115.9 (2)	C13—C14—H14	119.7
C10—O3—H3	109.5	C15—C14—H14	119.7
C6—C1—C2	120.1 (3)	C16—C15—C14	120.3 (3)
C6—C1—C7	117.1 (3)	C16—C15—H15	119.9
C2—C1—C7	122.8 (3)	C14—C15—H15	119.9
O1—C2—C3	118.3 (3)	C15—C16—C17	120.7 (3)
O1—C2—C1	124.5 (3)	C15—C16—H16	119.6
C3—C2—C1	117.2 (3)	C17—C16—H16	119.6
C4—C3—C2	121.6 (3)	C18—C17—C16	122.1 (3)
C4—C3—H3A	119.2	C18—C17—C12	118.3 (3)
C2—C3—H3A	119.2	C16—C17—C12	119.6 (3)
C3—C4—C5	120.2 (3)	C9—C18—C17	121.9 (3)
C3—C4—H4	119.9	C9—C18—H18	119.0
C5—C4—H4	119.9	C17—C18—H18	119.0
C6—C5—C4	119.8 (3)	Sn1—C19—H19A	109.5
C6—C5—Br1	121.4 (2)	Sn1—C19—H19B	109.5
C4—C5—Br1	118.7 (3)	H19A—C19—H19B	109.5
C5—C6—C1	120.9 (3)	Sn1—C19—H19C	109.5
C5—C6—H6	119.5	H19A—C19—H19C	109.5
C1—C6—H6	119.5	H19B—C19—H19C	109.5
N1—C7—C1	126.6 (3)	Sn1—C20—H20A	109.5
N1—C7—H7	116.7	Sn1—C20—H20B	109.5
C1—C7—H7	116.7	H20A—C20—H20B	109.5
O2—C8—N2	123.4 (3)	Sn1—C20—H20C	109.5
O2—C8—C9	119.1 (3)	H20A—C20—H20C	109.5
N2—C8—C9	117.5 (3)	H20B—C20—H20C	109.5
C18—C9—C10	119.4 (3)		
			
O1—Sn1—N1—C7	−5.4 (3)	Sn1—N1—C7—C1	−0.2 (5)
C19—Sn1—N1—C7	−97.1 (3)	C6—C1—C7—N1	−177.1 (3)
C20—Sn1—N1—C7	89.6 (3)	C2—C1—C7—N1	3.5 (5)
O2—Sn1—N1—C7	178.6 (3)	Sn1—O2—C8—N2	0.3 (4)
O1—Sn1—N1—N2	176.3 (2)	Sn1—O2—C8—C9	−179.4 (2)
C19—Sn1—N1—N2	84.6 (2)	N1—N2—C8—O2	0.0 (4)
C20—Sn1—N1—N2	−88.7 (2)	N1—N2—C8—C9	179.6 (3)
O2—Sn1—N1—N2	0.3 (2)	O2—C8—C9—C18	2.0 (4)
C7—N1—N2—C8	−178.8 (3)	N2—C8—C9—C18	−177.7 (3)
Sn1—N1—N2—C8	−0.3 (3)	O2—C8—C9—C10	−178.2 (3)
C19—Sn1—O1—C2	133.7 (3)	N2—C8—C9—C10	2.2 (5)
C20—Sn1—O1—C2	−97.1 (3)	C18—C9—C10—O3	178.5 (3)
O2—Sn1—O1—C2	21.7 (4)	C8—C9—C10—O3	−1.4 (5)
N1—Sn1—O1—C2	12.4 (3)	C18—C9—C10—C11	−2.7 (5)
O1—Sn1—O2—C8	−10.0 (4)	C8—C9—C10—C11	177.4 (3)
C19—Sn1—O2—C8	−122.1 (2)	O3—C10—C11—C12	−178.9 (3)
C20—Sn1—O2—C8	109.2 (2)	C9—C10—C11—C12	2.2 (5)
N1—Sn1—O2—C8	−0.3 (2)	C10—C11—C12—C13	179.4 (3)
Sn1—O1—C2—C3	166.5 (2)	C10—C11—C12—C17	0.2 (5)
Sn1—O1—C2—C1	−13.5 (5)	C11—C12—C13—C14	−177.7 (3)
C6—C1—C2—O1	−176.3 (3)	C17—C12—C13—C14	1.5 (5)
C7—C1—C2—O1	3.1 (5)	C12—C13—C14—C15	−1.0 (5)
C6—C1—C2—C3	3.7 (4)	C13—C14—C15—C16	0.4 (5)
C7—C1—C2—C3	−177.0 (3)	C14—C15—C16—C17	−0.3 (5)
O1—C2—C3—C4	178.9 (3)	C15—C16—C17—C18	−179.2 (3)
C1—C2—C3—C4	−1.1 (5)	C15—C16—C17—C12	0.9 (5)
C2—C3—C4—C5	−2.2 (5)	C11—C12—C17—C18	−2.1 (4)
C3—C4—C5—C6	2.8 (5)	C13—C12—C17—C18	178.7 (3)
C3—C4—C5—Br1	−174.0 (2)	C11—C12—C17—C16	177.8 (3)
C4—C5—C6—C1	−0.2 (5)	C13—C12—C17—C16	−1.4 (4)
Br1—C5—C6—C1	176.6 (2)	C10—C9—C18—C17	0.8 (5)
C2—C1—C6—C5	−3.1 (5)	C8—C9—C18—C17	−179.3 (3)
C7—C1—C6—C5	177.5 (3)	C16—C17—C18—C9	−178.3 (3)
N2—N1—C7—C1	178.1 (3)	C12—C17—C18—C9	1.6 (5)

### Hydrogen-bond geometry (Å, °)

**Table:**

*D*—H···*A*	*D*—H	H···*A*	*D*···*A*	*D*—H···*A*
O3—H3···N2	0.84	1.88	2.611 (4)	144
